# Detailed statistical analysis plan for the target temperature management after out-of-hospital cardiac arrest trial

**DOI:** 10.1186/1745-6215-14-300

**Published:** 2013-09-17

**Authors:** Niklas Nielsen, Per Winkel, Tobias Cronberg, David Erlinge, Hans Friberg, Yvan Gasche, Christian Hassager, Janneke Horn, Jan Hovdenes, Jesper Kjaergaard, Michael Kuiper, Tommaso Pellis, Pascal Stammet, Michael Wanscher, Matt P Wise, Anders Åneman, Jørn Wetterslev

**Affiliations:** 1 Department of Anesthesia and Intensive Care, Helsingborg Hospital, Södra Vallgatan 5, Helsingborg, 253 87, Sweden; 2 Department of Clinical Sciences, Lund University, Getingevägen 5, Lund, Sweden Lund, 221 85, Sweden; 3 Copenhagen Trial Unit, Centre of Clinical Intervention Research, Copenhagen University Hospital Rigshospitalet, Blegdamsvej 9, Copenhagen, 2100, Denmark; 4 Department of Neurology, Skåne University Hospital, Getingevägen 5, Lund, 221 85, Sweden; 5 Department of Cardiology, Skåne University Hospital, Getingevägen 5, Lund, 221 85, Sweden; 6 Department of Anesthesia and Intensive Care, Skåne University Hospital, Getingevägen 5, Lund, 221 85, Sweden; 7 Department of Intensive Care, Geneva University Hospital, 4 Rue Gabrielle-Perret-Gentil, Geneva, 1211, Switzerland; 8 The Heart Centre, Department of Cardiology, Copenhagen University Hospital Rigshospitalet, Blegdamsvej 9, Copenhagen, 2100, Denmark; 9 Department of Intensive Care, Academic Medical Center, Postbus 22700, Amsterdam, NL-1100 DE, Netherlands; 10 Department of Anaesthesia, Oslo University Hospital, Rikshospitalet, Songsvannsvejen 20, Oslo, 27, Norway; 11 Intensive Care Unit, Leeuwarden Medical Centrum, Borniastraat 38, Leeuwarden, NL8934 AD, Netherlands; 12 Intensive Care Unit, Santa Maria degli Angeli, Via Montereale 24, Pordenone, 33170, Italy; 13 Department of Anesthesia and Intensive Care, Centre Hospitalier, de Luxembourg, 4 rue Nicolas Ernest Barblé, Luxembourg, L 1210, Luxembourg; 14 Adult Critical Care, University Hospital of Wales, Heath Park, Cardiff, CF144XW, UK; 15 Department of Intensive Care, Liverpool Hospital, Sydney, Locked Bag 7103, Liverpool BC, NSW, 1871, Australia

**Keywords:** Cardiac arrest, Induced hypothermia, Mortality, Neurological function, Targeted temperature management, Randomized clinical trial, Statistical analysis plan

## Abstract

**Background:**

Animal experimental studies and previous randomized trials suggest an improvement in mortality and neurological function with temperature regulation to hypothermia after cardiac arrest. According to a systematic review, previous trials were small, had a risk of bias, evaluated select populations, and did not treat hyperthermia in the control groups. The optimal target temperature management (TTM) strategy is not known. To prevent outcome reporting bias, selective reporting and data-driven results, we present the a priori defined detailed statistical analysis plan as an update to the previously published outline of the design and rationale for the TTM trial.

**Methods:**

The TTM trial is an investigator-initiated, multicenter, international, randomized, parallel-group, and assessor-blinded clinical trial of temperature management in 950 adult unconscious patients resuscitated after out-of-hospital cardiac arrest of a presumed cardiac cause. The patients are randomized to a TTM of either 33°C or 36°C after return of spontaneous circulation. The primary outcome is all-cause mortality at maximal follow-up (until end of the trial and a minimum of 180 days). The main secondary outcomes are the composite outcome of all-cause mortality and poor neurological function (Cerebral Performance Category (CPC) 3 and 4, and modified Rankin Scale (mRS) 4 and 5) at hospital discharge and at 180 days; and assessment of safety and harm: bleeding, infections, electrolyte and metabolic disorders, seizures, cardiac arrhythmia, and renal replacement therapy.

**Conclusion:**

The TTM trial investigates potential benefit and harm of two target temperature strategies, both avoiding hyperthermia in a large proportion of the out-of-hospital cardiac arrest population.

**Trial registration:**

ClinicalTrials.gov identifier: NCT01020916

## Introduction

The target temperature management (TTM) trial is a randomized, parallel-group, assessor-blinded clinical trial, and is the largest trial to date of out-of-hospital post-cardiac arrest treatment and temperature management in the intensive care setting.

To prevent outcome reporting bias and data-driven analysis results, the International Conference on Harmonisation (ICH) of Good Clinical Practice (GCP) and others have recommended that clinical trials should be analyzed according to a pre-specified plan [1]. Leading experts in the critical care community have advocated that this should not only be a recommendation but rather a prerequisite [2]. Here, we describe the statistical analysis plan that has been finalized while data collection in the TTM trial still is on-going, and to which all data analyses in the main publication of the TTM trial results will adhere. The steering group of the TTM trial unanimously approved the statistical analysis plan on 3 December 2012, patient recruitment of 950 patients was completed on 10 January 2013, and the final follow-up was performed on 9 July 2013, after which the database was locked. The statistical analysis plan was published on ClinicalTrials.gov before last data entry and before data analysis was commenced.

### Trial overview

The TTM trial is a multicenter, international, outcome assessor-blinded, parallel group, randomized clinical trial (RCT) comparing two strict target temperature regimens of 33°C and 36°C. The population is adult patients, who have sustained return of spontaneous circulation and remain unconscious after out-of-hospital cardiac arrest on admission to hospital. The study background, design, and rationale have been previously published [3,4]. In brief, the induction of mild induced hypothermia (32°C to 34°C) has become an international standard for unconscious survivors of out-of-hospital cardiac arrest, being embraced by the European Resuscitation Committee, American Heart Association, and International Liaison Committee on Resuscitation, among others. The rationale for this therapy is largely based on the results of two RCTs [5,6], both reporting a substantial benefit of hypothermia. However, a recent systematic review and meta-analysis concluded that there was a lack of conclusive evidence supporting the use of mild hypothermia following cardiac arrest, and the quality of evidence was low using the Grading of Recommendations, Assessment, Development and Evaluation (GRADE) system [3]. As previous trials had not accounted for the presence of fever in control groups, the rationale for the TTM study was to compare mild induced hypothermia (33°C) with controlled normothermia (36°C).

The TTM trial protocol (current version 3.3) has been available online at http://www.ttm-trial.org since the start of the trial. The trial is registered at ClinicalTrials.gov (NCT01020916), and is endorsed by the European Clinical Research Infrastructure Network and the Scandinavian Critical Care Trials Group.

The trial was carried out in compliance with the Helsinki declaration and was approved by the ethical committees in each participating country: Australia: Health Ethics Review Committee Protocol No X11-0150 & HREC/11/RPAH/216 – “GI-CCT886; Czech Republic: Ethics committee of the General University Hospital of Prague, c/j 193–11 S 17.2.2011; Denmark: De vitenskabsetiske Komiteer i Region Hovedstaden, H-1-2010-059; Italy: Comitato Etico Indipendente, Hospedaliera S Maria degli Angeli Pordenone, No 9; Luxembourg: Comité National d’Ethique de Recherche CNER No 201007/05 Ver 1.0

The Netherlands: Medisch Etische Toetsingscommissie MEC 10/107 # 10.17.0921; Norway: Regional komité for medisinsk och helsefaglig forskningsetikk Sør-øst C Ref 2010/384; Sweden: Regional Ethical Review Board Lund, Protocol 2009/6 Dnr 2009/324 (TTM-Trial); Switzerland: Comité d’Ethique de Recherche CER 10–254 (NAC 10–088); United Kingdom: Cardiff and Vale Research Review Service, Project ID 10/AIC/4927, Research Ethics Committee for Wales: 10/MRE09/41.

### Objective

The primary aim of the TTM trial is to compare the effects of two strict target temperature protocols for the first 36 hours of hospital stay after resuscitation from out-of-hospital cardiac arrest (4 hours for achieving the target temperature, 24 hours of maintenance of target temperature, and 8 hours of rewarming). The null hypothesis is that there is no difference in survival until the end of the trial (180 days from randomization of the last patient) with a target temperature of 33°C compared to 36°C. To demonstrate or reject a hazard ratio difference of 20% between the groups, equivalent to approximately 1 month of difference in median survival time assuming proportional hazards in the groups during the observation time, a sample size of 900 patients would be necessary with a type I error risk of 5% and a type II error risk of 10%. To allow for patients lost to follow-up, the target population is set to 950 patients.

### Stratification and design variables

The only stratification variable used is trial site (hospital). Pre-defined design variables allowing for an adjusted analysis of the primary outcome and pre-defined subgroup analyses are: age, gender, first presenting cardiac rhythm (shockable or non-shockable), duration of cardiac arrest, and presence of shock at admission.

### Definition of the efficacy variables

The outcomes are defined as primary, secondary, and exploratory (tertiary in the trial protocol). Only primary and secondary outcomes will be analyzed for the first published report of the TTM trial due to the complexity of the exploratory outcomes, and thus a need for separate publications.

### Primary outcome

The primary outcome is survival until end of trial, which will be 180 days from randomization of the last patient.

### Secondary outcomes including adverse events

The main secondary outcomes are the composite outcomes of: 1) poor neurological function defined as Cerebral Performance Category (CPC) 3 or 4, or death (CPC 5); and 2) poor neurological function defined as modified Rankin Scale (mRS) 4 or 5, or death (mRS 6) evaluated at 180 days (± 14 days) from randomization. The number of study participants in each category of CPC and mRS will be reported separately.

The following adverse events are included in the secondary outcomes: bleeding, infection, electrolyte and metabolic disorders, cardiac arrhythmia, myoclonic or tonic-clonic seizures, and renal replacement therapy. The full list of adverse events is displayed in Table 1.

**Table 1:** Adverse events reported day 1 to 7 in the ICU

**Adverse event**	**Definition**
Renal replacement therapy	Continuous or intermittent
Seizures	Myoclonic and tonic-clonic
Bleeding	Uncontrolled bleeding (defined as the need for transfusion with 1 unit of blood per 10 kg/h), intracranial, intraspinal, intraocular, intra-articular, pericardial, gastrointestinal, tracheal, oral cavity, nose, genital, and bleeding from insertion sites
Infection	Pneumonia, severe sepsis, septic shock, and other serious infection
Cardiac arrhythmia	Atrial fibrillation, atrial flutter, tachycardia, bradycardia, ventricular tachycardia, ventricular fibrillation, and cardiac arrest mandating cardiopulmonary resuscitation (CPR)
Electrolyte and metabolic disorders	Hypokalemia (<3.0 mmol/L), hypomagnesemia (<0.7 mmol/L), hypophosphatemia (<0.7 mmol/L), and hypoglycemia (<3.0 mmol/L)

Other secondary outcomes are CPC at ICU and hospital discharge, and best reported CPC during entire trial period.

### Exploratory outcomes

Neurological function at 180 days will be defined with CPC, mRS, Informant Questionnaire on Cognitive Decline in the Elderly (IQCODE) (questionnaire directed to a relative or close acquaintance), mini-mental state examination (MMSE), and two simple questions: 1a) In the last 2 weeks, did you require help from another person for your everyday activities? (If yes: 1b) Is this a new situation following the heart arrest?); and 2) Do you feel you have made a complete mental recovery after your heart arrest? The neurological function tests will be supplemented with a questionnaire exploring quality of life defined with the short-form 36 (SF-36) [4].

### Data points

#### Baseline variables

The baseline variables will be:

1. Sex

2. Age

3. Comorbidities (only reported if the frequency is above or equal to 5% in any of the intervention groups; pre-morbid CPC will be reported regardless of the frequency)

3.a. Chronic heart failure (New York Heart Association (NYHA) Class 3 or worse)

3.a. Previous acute myocardial infarction (AMI)

3.a. Ischemic heart disease

3.a. Previous cardiac arrhythmia

3.a. Previous cardiac arrest

3.a. Arterial hypertension

3.a. Previous transient ischemic attack or stroke

3.a. Epilepsy

3.a. Diabetes mellitus

3.a. Asthma or chronic obstructive pulmonary disease (COPD)

3.a. Chronic hemodialysis or peritoneal dialysis

3.a. Hepatic cirrhosis

3.a. Hematological malignancy

3.a. Other malignancy

3.a. AIDS

3.a. Alcoholism

3.a. Intravenous drug abuse

3.a. Other immunodeficiency

3.a. Pre-morbid CPC

4. Previous percutaneous coronary intervention (PCI)

5. Previous coronary bypass grafting

6. Previous valvular surgery

7. Implantable cardioverter-defibrillator (ICD) and/or pacemaker

8. Pre-hospital variables

8.a. Location of cardiac arrest

8.a. Bystander witnessed arrest

8.a. Bystander cardiopulmonary resuscitation (CPR)

8.a. First monitored rhythm at arrival of emergency medical service

8.a. Use of active compression-decompression device

8.a. Time from cardiac arrest to start of basic life support

8.a. Time from cardiac arrest to start of advanced life support

8.a. Time from cardiac arrest to return of spontaneous circulation

9. Data on admission

9.a. First measured temperature (tympanic)

9.a. Glasgow Coma Scale (GCS) (combined score)

9.a. pH

9.a. Lactate

9.a. Shock on admission

9.a. Acute ST-elevation infarction or new left bundle branch block (LBBB).

### Intervention period variables

Core temperature primarily measured in the urinary bladder will be reported per hour during the 36 hours of the intervention period.

### Neurological prognostication and withdrawal of care

The number and proportion of patients still comatose at 72 hours after the end of the intervention period that underwent neurological prognostication by a blinded physician will be reported. The number of patients who did not survive until neurological prognostication and their presumed cause of death, including limitations in care and reasons for that will be recorded. The number of patients with electroencephalogram (EEG), somatosensory evoked potentials (SEPs), magnetic resonance imaging (MRI), and computed tomography (CT) of the head will also be reported.

### Concomitant cardiological treatments

The number of patients receiving coronary angiography, PCI, and coronary bypass grafting, divided in three time groups (immediately after admission, during intervention or when sedated in the ICU, and after regaining consciousness) will be reported. The number of patients receiving intra-aortic balloon pump (IABP), other mechanical assist device, temporary pacemaker, permanent pacemaker, and ICD will also be reported.

### Other descriptive variables

The number of days in the ICU and days on mechanical ventilation during the index ICU admission and days in hospital within the index admission will be reported.

### General analysis principles

The general analysis principles will be:

1. Analyses will be conducted according to the modified intention-to-treat principle (ITT) [7] if not otherwise stated.

2. All tests of significance will be two-sided with a maximal type I error risk of 5%.

3. The primary analyses of primary and secondary outcomes will be those of the modified ITT population adjusted for the protocol specified stratification variable [8] and if necessary using data sets generated using multiple imputations. An unadjusted analysis and an analysis adjusting for both stratification and pre-defined design variables will be carried out as sensitivity analyses. Other analyses may also be performed using, for example, a slightly different population. If the results of these analyses are not consistent with the primary analyses this will be discussed. Nevertheless, the conclusions of the study will still be those based on the primary analyses.

4. The tests for interaction between the intervention and each design variable used to identify subgroups are exploratory.

5. Risks will be reported as hazard ratios or risk ratios with 95% confidence interval (CI) or with limits as stated in point 6.

6. If there is data missingness for a specified primary or secondary outcome of less than 5% a complete case analysis without imputing missing values will be performed. If there is a missingness of more than 5% Little’s test will be performed. If the test indicates that the complete case data set is a random sample we will continue without imputing missing values and analyze the complete cases. If Little’s test indicates that the data set of complete cases is not a random sample of the total data set we will report the point estimates and their 95% confidence limits by applying a worst/best scenario imputation for the missing values. If the worst/best case analyses allow for the same conclusion we will not perform multiple imputations. However, if the worst/best case imputation provides different conclusions, multiple imputations will be performed, creating ten imputed data sets under the assumption of missingness at random. The result of the trial will be the pooled intervention effect and 95% CI of the analyses of the data sets after multiple imputations. The unadjusted, non-imputed analysis will also be made available.

Primarily the observed *P* values of the primary and five secondary outcomes will be presented. However, multiplicity, a possible reason for spurious statistically significant *P* values, may be a problem when the results of several outcomes are presented. We therefore want to present a supplemental analysis with the results of *P* values adjusted for multiplicity according to the fallback procedure [9]. The *P* values adjusted for multiplicity will be presented and discussed in relation to the unadjusted *P* values. This adjustment may be needed to control the overall probability of a type I error (rejection of a null hypothesis that is actually true) and keep the familywise error rate (FWER) below 0.05 as required by most regulatory agencies. This will be undertaken by specifying the weights of the hypotheses assigned to them according to their importance. The sequence in which the hypotheses will be tested and their individual weights (in parentheses) will be: primary outcome (0.50), first secondary outcome (0.25), second secondary outcome (0.0625), third secondary outcome (0.0625), fourth secondary outcome (0.0625), and fifth secondary outcome (0.0625). The multiplicity problem is addressed further in the Discussion section.

### Statistical analyses

#### Trial profile

**Figure 1:**
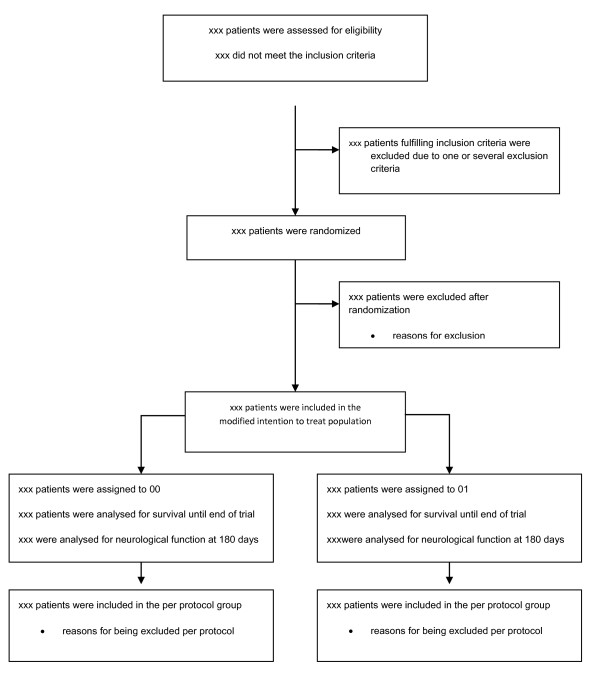
CONSORT flow diagram.

The flow of study participants will displayed in a Consolidated Standards of Reporting Trials (CONSORT) diagram as shown in Figure 1[10]. The number of screened patients who fulfilled study inclusion criteria, and the number included in the primary and secondary analyses as well as all reasons for exclusions in primary and secondary analyses will be reported.

### Primary outcome

Frequencies and percentages per group as well as hazard ratios with 95% CI will be reported. The primary outcome will be analyzed using Cox regression with adjusted variables. The proportional hazards assumption across treatment groups will be checked by testing if there is an interaction between intervention and time, and by plotting cumulative hazard functions for intervention groups.

The first analysis of the primary outcome, adjusted for the stratification variable, will be on the patients that met the inclusion criteria and did not meet the exclusion criteria at time of randomization. Patients who did not meet the inclusion criteria and did not receive the intervention (temperature management) and were erroneously randomized will be excluded according to the modified ITT principle.

The second analysis of the primary outcome will be on patients that met the inclusion criteria and did not meet the exclusion criteria and did not have any major protocol violations (per-protocol analysis).

The third analysis of the primary outcome will be an analysis adjusted for both the stratification variable and the design variables.

The above analyses will be repeated with sites grouped as a variable indicating whether the patient has been allocated by the two sites having allocated most patients or one of the other sites (which would be approximately one quarter of the trial population).

### Secondary outcomes including adverse events

Frequencies and percentages per group as well as risk ratios with 95% CI will be reported. A standard chi-squared test will be used to assess the effect of treatment on binary and categorical outcomes. For the adjusted primary analyses logistic regression analysis will be used. The Wilcoxon-Mann–Whitney test will be used for continuous outcomes. There will only be reported significance testing on the composite outcomes of mortality and poor neurological outcome versus survival with good neurological outcome; not on the individual sub-scores of CPC and mRS. For adverse events there will be a chi-squared test on having one or more adverse events versus having no adverse events. If there is a significant difference between treatment groups in occurrence of adverse events we will try to delineate which events drive this difference. However, we acknowledge the low power for performing analyses in this case.

### Characteristics of patients with baseline comparisons

The description of baseline characteristics listed above will be presented by treatment group. Discrete variables will be summarized by frequencies and percentages. Percentages will be calculated according to the number of patients where data are available. Where values are missing, the actual denominator will be stated.

Continuous variables will be summarized using standard measures of central tendency and dispersion, using either mean ± SD for data with normal distribution or median and interquartile range for non-normally distributed data.

### Intervention period variables

The mean values of the actual measured temperature in the two intervention groups will be displayed in a graph with mean ± 2 SD.

### Neurological prognostication and withdrawal of care, concomitant cardiological treatments, and other descriptive variables

The description of baseline characteristics listed above will be presented by treatment group without significance testing. Discrete variables will be summarized by frequencies and percentages. Percentages will be calculated according to the number of patients where data are available. Where values are missing, the actual denominator will be stated.

Continuous variables will be summarized using standard measures of central tendency and dispersion, using either mean ± SD for data with normal distribution or median and interquartile range for non-normally distributed data.

### Outline of figures and tables

The first figure will be a CONSORT flow chart as specified in Figure 1. The second figure will be a temperature graph for the two groups with hours 0 to 36 on the x-axis and mean temperature ± 2 SD on the y-axis. The third figure will be a Kaplan-Meier plot of survival in the two groups during the trial period (32 months). The fourth figure will be a forest plot of intervention effects stratified for the design variables: age dichotomized around the median, gender, duration of cardiac arrest dichotomized around the median, initial cardiac rhythm (shockable or non-shockable), and presence or absence of cardiogenic shock at admission to hospital.

## Discussion

With this statistical analysis plan we present the different analyses in the main publication of the TTM trial in order to avoid risks of outcome reporting bias and data-driven results. Of the pre-specified results in the trial we choose to report only primary and secondary outcomes in the main publication, because of the complexity of the detailed neurological outcomes and quality of life that constitutes the exploratory outcomes, necessitating separate publications.

We would like to emphasize that the main secondary outcome, the composite outcome of poor neurological function and mortality at 180 days after cardiac arrest, will be of great significance in a situation where the primary outcome measure shows a neutral result. No significant difference in mortality, but a clear difference in functional outcome, or opposing outcomes, will have implications for the interpretation of the trial. Survival is an outcome with a low risk of bias and not prone to competing risks. Earlier trials and registry data indicate that a smaller sample size is needed to show the same risk reduction when the composite outcome of mortality and poor neurological function is used (compared to mortality/survival). This was the basis for the order of the outcomes. The composite outcome of poor neurological function and mortality will benefit from an increased power with respect to the possibility of finding or rejecting a significant signal when the trial is powered for survival, which would require a larger sample size.

### Comments on the multiplicity problem

There are one primary and five secondary outcomes to be assessed. The primary outcome is survival. The secondary outcomes are:

1) neurological (CPC), binary quantity; 2) neurological (mRS), binary quantity; 3) adverse event, binary quantity; 4) CPC measured at specified point in time, binary quantity; and 5) best cerebral performance during specified period, binary quantity.

Thus, there are six significance tests. These have to be adjusted for multiplicity to control the probability of a type I error (rejection of a null hypothesis that is true). One way to diminish this risk would be to deal with the six outcomes as one group using a data-driven adjustment of the *P* values. The most powerful procedure based on the raw *P* values is probably that of Hommel [9].

An alternative (the fixed sequence procedure) would be to specify the sequence of the hypotheses testing in advance (primary outcome, first secondary outcome, second secondary outcome, third secondary outcome, fourth secondary outcome, and fifth secondary outcome). In this latter case, no multiplicity adjustment will be needed. Each test will then be performed at the 0.05 level of significance in the specified order. However, as soon as a test is non-significant the remaining null hypotheses will be accepted without test. For instance, if the primary outcome and the first secondary outcome are significant at the 0.05 level and the second secondary outcome (neurological function measured with mRS) is insignificant, the null hypotheses corresponding to the third, fourth, and fifth secondary outcomes will be accepted without test.

A third approach is the so-called fallback procedure where the fixed hypothesis testing sequence is also used. However, if a test is insignificant, the procedure does not stop but the next hypothesis is tested at a reduced level of significance. This procedure also allows the hypotheses to be weighted according to their importance and likelihood of being rejected.

It appears from Table 2 that Hommel’s procedure is sensitive to the *P* values of the last three tests, while the fallback procedure is not. Since the first and second of the secondary outcomes will most likely produce similar *P* values, it would be logical to place most of the weights on the primary and first secondary outcome.

**Table 2:** **Examples of adjustment of raw *P *values using fallback procedure and Hommel’s procedure**

**Weight**	**Raw *P *value (example 1)**	**Fallback procedure**	**Hommel’s procedure**	**Raw *P *value (example 2)**	**Fallback procedure**	**Hommel’s procedure**
0.5	0.030	0.060	0.055	0.030	0.060	0.030
0.25	0.010	0.040	0.050	0.010	0.040	0.0225
0.0625	0.015	0.048	0.055	0.015	0.048	0.030
0.0625	0.055	0.0629	0.055	0.001	0.016	0.0040
0.0625	0.055	0.0629	0.055	0.001	0.016	0.0040
0.0625	0.055	0.0629	0.055	0.001	0.016	0.0040

Based on these considerations the analyses in the TTM trial will be presented with unadjusted *P* values as well as adjusted for multiplicity using the fallback procedure.

## Conclusion

To conclude, this article describes the principles of analysis used in the TTM trial for the first publication of the main outcomes. Our approach aims to minimize the risk of data-driven results and outcome reporting bias.

## Abbreviations

AMI: Acute myocardial infarction; CI: Confidence interval; CONSORT: Consolidated standards of reporting trials; COPD: Chronic obstructive pulmonary disease; CPC: Cerebral performance category; CPR: Cardiopulmonary resuscitation; CT: Computed tomography; EEG: Electroencephalogram; FWER: Familywise error rate; GCP: Good clinical practice; GCS: Glasgow coma scale; GRADE: Grading of recommendations, assessment, development and evaluation; IABP: Intra-aortic balloon pump; ICD: Implantable cardioverter-defibrillator; ICH: International conference on harmonisation; IQCODE: Informant questionnaire on cognitive decline in the elderly; ITT: Intention-to-treat; LBBB: Left bundle branch block; MMSE: Mini-mental state examination; MRI: Magnetic resonance imaging; mRS: modified rankin scale; NYHA: New york heart association; PCI: Percutaneous coronary intervention; RCT: Randomized clinical trial; SEP: Somatosensory evoked potentials; SF-36: Short-form 36; TTM: Target temperature management.

## Competing interests

The authors declare no financial or non-financial competing interests.

## Authors’ contributions

NN, PW, and JW proposed the statistical analysis plan. NN drafted the manuscript. NN, TC, DE, HF, YG, CH, JHor, JHov, JK, MK, TP, PS, MW, MPW, AÅ, and JW participated in the design and coordination of the trial. NN, PW, TC, DE, HF, YG, CH, JHor, JHov, JK, MK, TP, PS, MW, MPW, AÅ, and JW read, amended and approved the statistical analysis plan and the final manuscript. All authors read and approved the final manuscript.
